# Scientific Slang

**Published:** 1902-03

**Authors:** 


### EDITORIAL NOTES AND COMMENTS.

The Business office of THE JOURNAL-RECORD is No. 64 Marietta Street.

The Editorial office is 318-319-320 The Prudential.

Address all Business communications to Dr. M. B. Hutchins, Mgr.

Make remittances payable to THE ATLANTA JOURNAL-RECORD OF MEDICINE.

On matters pertaining to the Editorial and Original Communications address Dr. Bernard Wolff, Atlanta.

Reprints of original articles will be furnished at cost price. Requests for the same should always be made *on the manuscript*.

We will present, post-paid, on request, to each contributor of an original article, twenty (20) marked copies of THE JOURNAL-RECORD containing such article.

### SCIENTIFIC SLANG.

ONE of the principal aims of science is the simplification of things. The simpler the science the more exact it is apt to be. This rule or tendency holds good in words as well as in things, or should hold good. Yet nowhere is science more burdened than in its nomenclature. The Germanic style of piling up words, particularly in the department of organic chemistry is almost from its unmanageableness reduced to an absurdity. Synthetic compounds in which every element of the synthesis must be philologically respected look well enough on paper with hyphens introducd for purposes of respiration, but they present obstacles to conversation. Another tyranny under which the language of science groans is that of attaching someone's name to a certain method or procedure of whatever character. To properly understand the eponymic reference one's knowledge must be positively encyclopedic and his memory prodigious, otherwise it is meaningless and so much gibberish. Take the following paragraph: "The total nitrogen was estimated by the Kjeldahl method; the urea by the Mörner-

Sjöqvist method; the uric acid by the Salkowski-Ludwig method, as taught by Salkowski, and the purin bases by the new Salkowski method." This was taken from a medical journal intended for the general practitioner and probably conveys about as much information to him as though it were an inscription on a winged bull from Babylon. This obscuring under a proper name is more the outcome of human vanity than of scientific convenience. It would be just as easy to give some catch-word or clue to the meaning with the proper name bracketed, if need be, and thus avoid a tedious and reluctant search by the reader. The French are prime offenders in this particular. Every Frenchman strives to render himself immortal by having his name associated with some anatomical process, physiological reaction or disease and the like, though fortunately few of these Gallic conceits survive translation. The pace of medical progress is almost enough to give one vertigo to attempt to follow it, and obstructions of whatever character are not welcomed and are fit objects for removal.

---

DR. W. E. FITCH, founder, and for many years editor and business manager of *The Georgia Journal of Medicine and Surgery*, published at Savannah, Ga., has sold his interest in the publication to his former associate and co-editor, Dr. St. J. B. Graham, who becomes editor and sole proprietor. The *Journal*, under Dr. Fitch's editorial management, from the appearance of the first issue, merited the support of the profession, and gradually, year after year, made for itself a place among the best medical periodicals of this country. The Doctor will devote his entire attention to the practice of his profession in Savannah, Ga.

